# The effects of low-dose Nepsilon-(carboxymethyl)lysine (CML) and Nepsilon-(carboxyethyl)lysine (CEL), two main glycation free adducts considered as potential uremic toxins, on endothelial progenitor cell function

**DOI:** 10.1186/1475-2840-11-90

**Published:** 2012-08-01

**Authors:** Jinzhou Zhu, Ke Yang, Yajun Jing, Run Du, Zhenbin Zhu, Lin Lu, Ruiyan Zhang

**Affiliations:** 1 Department of Cardiology, Rui Jin Hospital, School of Medicine, Shanghai Jiao Tong University, Shanghai, 200025, China; 2 Institute of Cardiovascular Diseases, School of Medicine, Shanghai Jiao Tong University, Shanghai, 200025, China

**Keywords:** Endothelial progenitor cells, Mitogen-activated protein kinases, Nϵ-(carboxyethyl)lysine, Nϵ-(carboxymethyl)lysine, Uremic toxins

## Abstract

**Background:**

Patients with chronic kidney disease (CKD) are at high risk of cardiovascular disease (CVD). Endothelial progenitor cell (EPCs) dysfunction plays a key role in this pathogenesis. Uremic retention toxins have been reported to be in associated with EPC dysfunction. Advanced glycation end-products (AGEs) free adducts, including Nepsilon-(carboxymethyl)lysine (CML) and Nepsilon-(carboxyethyl)lysine (CEL), are formed by physiological proteolysis of AGEs and released into plasma for urinary excretion. They are retained in CKD patients and are considered to be potential uremic toxins. Though AGEs have been demonstrated to impair EPC function in various ways, the effect of AGE free adducts on EPC function has not been studied. Thus, we examined the role of CML and CEL in the regulation of growth-factor-dependent function in cultured human EPCs and the mechanisms by which they may affect EPC function.

**Methods:**

Late outgrowth EPCs were incubated with different concentrations of CML or CEL for up to 72 hours. Cell proliferation was determined using WST-1 and BrdU assays. Cell apoptosis was tested with annexin V staining. Migration and tube formation assays were used to evaluate EPC function.

**Results:**

Though CML and CEL were determined to have anti-proliferative effects on EPCs, cells treated with concentrations of CML and CEL in the range found in CKD patients had no observable impairment on migration or tube formation. CML and CEL did not induce EPC apoptosis. The reduced growth response was accompanied by significantly less phosphorylation of mitogen-activated protein kinases (MAPKs).

**Conclusions:**

Our study revealed that CML and CEL at uremic concentrations have low biological toxicity when separately tested. The biologic effects of AGE free adducts on the cardiovascular system merit further study.

## Background

Patients with chronic kidney disease (CKD) are at high risk of cardiovascular disease (CVD) [1,2]. Endothelial dysfunction plays a key role in this pathogenesis [3-5]. Recent studies have repeatedly shown that decreased numbers of endothelial progenitor cells (EPCs), as in the case of CKD patients, is associated with an increase in the number of cardiovascular events [6-9]. The factors that cause such EPC-linked pathology in CKD patients are still not well understood.

CKD is attributed to the progressive retention of a large number of compounds which, under normal conditions, are excreted by the healthy kidneys [10]. These compounds are called uremic retention solutes, or when they interact negatively with biological functions, uremic toxins. Protein-bound uremic toxins constitute a heterogeneous group of compounds which are difficult to be removed by dialysis [11]. Inadequate removal of protein-bound uremic toxins has been reported as a primary cause of CVD in CKD patients [12,13]. Several protein-bound uremic toxins have been associated with EPC dysfunction [14,15].

Advanced glycation end-products (AGEs) are the result of a chain of chemical reactions after an initial glycation reaction. AGEs were evaluated in CKD patients and considered as candidate protein-bound uremic toxins [16,17]. The negative impact of AGEs on EPCs has been demonstrated repeatedly [18-21]. Physiological proteolysis of AGEs forms protein glycation free adducts that are released into the plasma for urinary excretion. Inefficient elimination of these free adducts in uremia may lead to their accumulation [22,23]. Patients with mild chronic renal failure not requiring dialysis had plasma glycation free adduct concentrations increased up to five-fold the normal level, associated with a decline in renal clearance. Patients on peritoneal dialysis (PD) had plasma glycation free adducts levels up to 18 times higher than normal, and those treated with hemodialysis (HD) up to 40 times higher [23].

Nepsilon-(carboxymethyl)lysine (CML) and Nepsilon-(carboxyethyl)lysine (CEL) are two main glycation free adducts in CKD patients. The European Uremic Toxin (EUTox) Work Group defined CML as protein-bound uremic toxins and CEL as potential toxins to be considered in the future [17,24]. Clinical studies have found a correlation between CML levels and mortality in hemodialysis patients [25]. CML has been associated with increased aortic pulse wave velocity in adults [26]. Animal studies show CML accelerates atherosclerotic calcification in diabetes [27]. *In vitro* studies show CML-albumin and CEL-albumin stimulate VCAM-1 expression in endothelial cells and activates leukocyte responses [28,29]. For these reasons, we decided to investigate the effects of CML and CEL on EPC function.

In the present study, we hypothesized that CML and CEL might have a deleterious impact on EPC function. We investigated the role of CML and CEL in the regulation of growth-factor-dependent function among cultured human EPCs and the mechanisms by which they might affect EPC function.

## Materials and methods

This study was approved by the Institutional Review Board of Shanghai Jiaotong University School of Medicine.

### Reagents

CML and CEL were purchased from Santa Cruz Co. (CA, USA). They were dissolved in 0.9% sodium chloride to a final concentration of 1 mg/ml and served as stocking solutions. Endotoxin levels of CML and CEL were measured by end-point quantitative chromogenic assay using the Tachypleus Amebocyte Lysate kit (Houshiji, Xiamen, China). The final preparation of these two compounds contained undetectable levels of endotoxin (detection sensitivity of 1 EU/μg).

### Culture and determination of human late outgrowth EPCs

Culture and determination of human late outgrowth EPCs were performed as previously reported [30]. Mononuclear cells (MNCs) were isolated from 20 ml human peripheral blood by Ficoll density-gradient centrifugation (1.077 g/ml Sigma, MO, USA). Recovered MNCs were then washed twice with phosphate buffered saline (PBS) and resuspended with Medium 199 (Gibco, MI, US) supplemented with 10% fetal bovine serum (FBS) and EGM-2 Single Quotes (Lonza, MD, USA). Cells were seeded on human fibronectin-coated (BD Biosciences, CA, USA) six-well plates and incubated in a 5% CO_2_ incubator at 37°C. Late EPCs colonies appeared after 2–4 weeks of culture. These cells were harvested and cultured for later experiments. Evaluations revealed cellular incorporation of acetylated LDL and UEA-1 binding affinity. Fluorescence-activated cell sorting (FACS) analysis showed that these cells were CD34-positive, CD31-positive, VEGFR2-positive, CD45-negative, and CD133-negative.

### EPC proliferation assays

The effects of CML and CEL alone on EPC proliferation were determined using two methods: (i) WST-1 assay kit (Roche Applied Science, Mannheim, Germany) and (ii) BrdU Labeling and Detection Kit (Calbiochem, NJ, USA). For the WST-1 assay, 5,000 cells per well were seeded in 96-well culture plates and incubated for 24 hours. CML at mean concentrations of 15, 46, 137, 412, 1235, 3704, 11111, 33333, and 100000 μg/L and CEL at mean concentrations of 46, 137, 412, 1235, 3704, 11111, 33333, 100000, and 300000 μg/L were added to the culture plates and incubated for 48 and 72 hours in a 5% CO2 incubator at 37°C. Sodium chloride solution served as a control. Ten microliters of cell proliferation assay reagent WST-1 was added to each well and incubated for 4 hours. Absorbance of 450 nm was measured by an enzyme linked immunosorbent assay (ELISA) reader (BioTek, VT, USA). For the BrdU assay, cells were incubated with BrdU and different concentrations of CML or CEL for 24 hours. Cellular incorporation of BrdU was measured by ELISA with a BrdU Labeling and Detection Kit, according to the manufacturer’s instructions.

### Tube formation assay

The tube formation capacity of EPCs was investigated using matrigel, as previously described [31]. Concentrations of CML and CEL similar to those found in CKD patients [25] were chosen to study. EPCs were incubated with 250, 500, and 1000 μg/L of CML or CEL for 72 hours. Controls received the same volume of sodium chloride solution. EPCs (2 × 10^4^ cells per well) were harvested and placed on a 96-well glass slide pre-coated with Matrigel (BD Bioscience, CA, USA). After 12 hours of incubation, six random high-power (100x) microscope fields were examined and graded by two investigators as follows: 0, separated individual cells; 1, cells begin to migrate and align; 2, capillary tubes visible, but no sprouting; 3, sprouting of new capillary tubes visible; 4, closed polygons begin to form; and 5, complex mesh-like structures develop. The average of these six fields was taken. Investigators were blinded to experiment protocol.

### EPC migration assay

The migratory function of EPCs was evaluated using a modified Boyden chamber (Transwell, Corning Inc., MA, USA) assay. In brief, EPCs were incubated using different concentrations of CML or CEL as described in the tube formation assay for 72 hours. EPCs (4 × 10^4^ cells per well) were then harvested, resuspended in 0.5% FBS, and placed in the upper chamber of 24-well transwell plates with a polycarbonate membrane (8 μm pores). VEGF (50 ng/ml) in medium was added to the lower chamber. After incubation for 12 hours, the membrane was washed briefly with hank’s balanced salt solution (HBSS). The upper side of the membrane was wiped gently with cotton wool. The membrane was then stained using Hochest 33342 (Invitrogen, CA, USA). Migration of EPCs was evaluated by measuring the area containing migrated cells as a percentage of the total area. Six random high-power (100x) microscope fields were examined and the average of these six fields was taken. The experiment was repeated three times.

### Cell apoptosis assay

The effect of CML or CEL on EPC apoptosis was determined with an Annexin V-Alexa Fluor 488 kit (Invitrogen, OR, USA). EPCs cultured on 6-well culture plates were treated with different concentrations of CML or CEL, as described in the tube formation assay. After 72 hours, cells were detached with trypsin, washed in M199 medium containing 10% FBS, centrifuged, and washed with ice-cold PBS. After centrifugation, the pellets were resuspended in binding buffer containing Alexa Fluor 488-annexin V and propidium iodide, mixed gently, and incubated on ice for 10 minutes in the dark. The samples were read using a FACSCalibur flow cytometer (Becton–Dickinson, CA, USA) at 494 nm. A 520 nm bandpass filter was used for Alexa Fluor 488 detection, and a greater than 600 nm filter was used for propidium iodide detection. The percentage of annexin-V-positive cells in the EPC population was determined using CellQuest Pro software (Becton–Dickinson, CA, USA). The experiment was repeated three times.

### Preparation of cell lysates and Western blotting

After EPCs were exposed to different concentrations of CML or CEL in complete medium 199 for up to 72 hours, they were rinsed twice with ice-cold PBS. Protein was extracted using ProteoJET Mammalian Cell Lysis Reagent (Fermentas, MD, USA). The protein content of cell lysates was separated on 10 or 12% gels using SDS-PAGE, and transferred to a PVDF membrane. Membranes were incubated overnight with the primary antibodies in Tris Buffered Saline with Tween-20 (TBST) and 5% bovine serum albumin (BSA) at 4°C. The membranes were washed three times with TBST and incubated for 1 hour at room temperature in horseradish peroxidase-conjugated goat anti-mouse or goat anti-rabbit secondary antibodies (1:7500 dilution in TBST containing 5% nonfat milk). The immunoreactive proteins were detected using an ECL Western blotting detection system (Millipore, MA, USA). Detection of GAPDH was used as the protein loading control. Each experiment was repeated three times. A representative blot is shown for each experiment.

### Statistical analysis

Data were analyzed using SPSS 13.0 software (SPSS, Inc. Chicago, USA). Results were expressed as mean ± standard deviation (SD). Statistically significant differences among different treatment groups at a single point in time were determined by non-parametric tests. Statistical significance was assumed for p<0.05.

## Results

### Effects of CML and CEL on EPC proliferation

**Figure 1:**
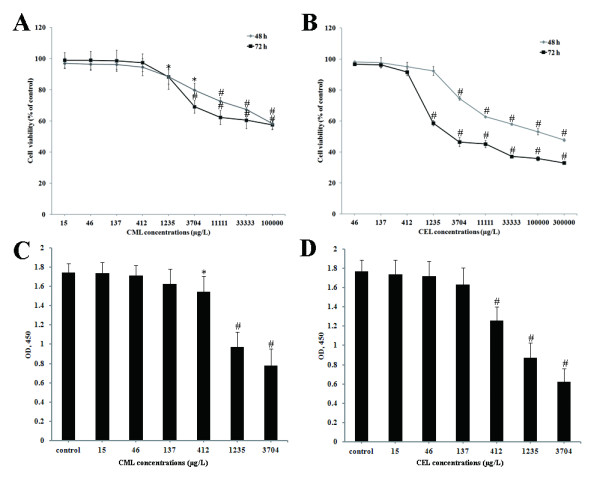
**Effects of CML and CEL on EPC proliferation. **(**A + B**) EPCs were incubated with different concentrations of CML or CEL alone and counted after 48 and 72 hours using the WST-1 method. (**C + D**) BrdU incorporation into EPCs incubated with different concentrations of CML or CEL for 24 hours were measured using enzyme-linked immunosorbent assay (ELISA). Data were expressed as mean ± SD of five independent experiments. *p<0.05 vs control, ^#^p<0.01 vs. control.

The effects of CML or CEL alone on EPC proliferation were first analyzed by WST-1 assay. Incubation of EPCs with different concentrations of CML or CEL for 48 and 72 hours both induced significant reductions in the number of adherent EPCs. This inhibition was dose-dependent (Figure 1A,B). The WST-1 results were confirmed by ELISA analyzing BrdU incorporation into cellular DNA, which showed CML and CEL inhibited EPC proliferation in a dose-dependently manner after 24 hours of incubation (Figure 1C,D).

### Effects of CML and CEL on EPC tube formation, migration, and apoptosis

**Figure 2:**
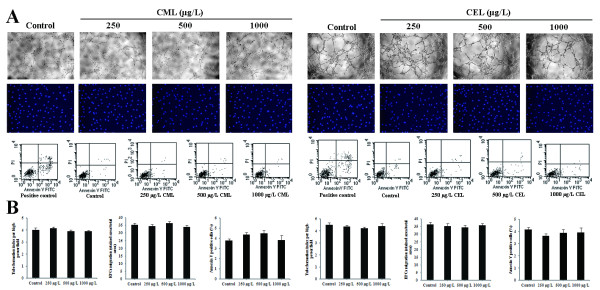
**Effects of CML and CEL on EPC tube formation, migration, and apoptosis. **EPCs were incubated with 250, 500, or 1000 μg/L of CML, CEL, or control for 72 hours and then tested. (**A**) Typical cell tube formation, migration, and apoptosis (100x). (**B**) Bar graph. No significant differences were observed between any of the different groups. Data were expressed as mean ± SD of three independent experiments.

CML or CEL alone at mean concentrations of 250, 500, and 1000 μg/L were chosen for studies of their effects on EPC tube formation, migration, and apoptosis. We first used a Matrigel model to examine whether CML or CEL alone would decrease EPCs ability to differentiate into capillary-like structures. Compared with control groups, CML and CEL had no observable effects on EPC tube formation. To determine the effects of CML or CEL alone on EPC migration, we counted cells moving through an insert filter in a chamber filled with medium 199 and 0.5% FBS. CML and CEL did not decrease EPC migration compared to the control group. The decreased numbers of EPCs after *ex vivo* incubation of CML or CEL may be the result of several factors. Previous studies demonstrated that AGEs could induce EPC apoptosis [19]. Thus, we determined whether CML or CEL could also induce EPC apoptosis using annexin V-FACS staining. Incubation of EPCs with different concentrations of CML or CEL for 72 hours did not induce cell apoptosis (Figure 2).

### Effects of CML and CEL on RAGE expression in EPCs

**Figure 3:**
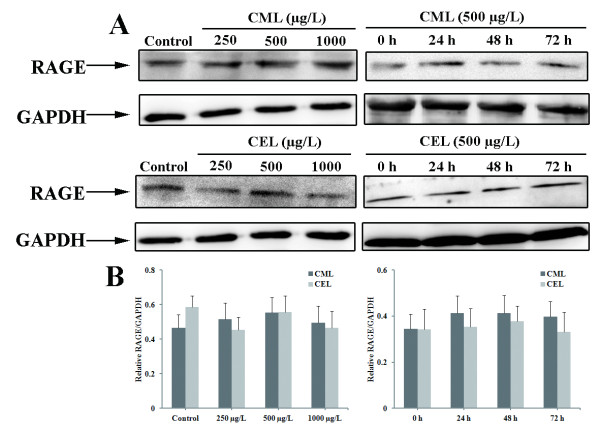
**Regulation of RAGE expressions in EPCs incubated with different concentrations of CML/CEL for different times. **(**A**) In the concentration-dependent experiment, EPCs were incubated with 250, 500, or 1000 μg/L of CML, CEL, or control for 72 hours. In the time-dependent experiment, EPCs were incubated with 500 μg/L of CML or CEL for 0, 24, 48, or 72 hours. Aliquots of cell lysate were then subjected to western blot analysis. (**B**) Bar graph. No CML or CEL-induced difference in RAGE expression was observed in EPCs. Results were expressed as mean ± SD of three independent experiments.

After observing the anti-proliferative effects of CML and CEL alone on EPCs, we next evaluated the expression of Receptor for advanced glycation end products (RAGE) in cells. RAGE is the main receptor through which AGE signaling is mediated [32]. In these experiments, EPCs were first incubated with different concentrations of CML or CEL for 72 hours. No CML and CEL-induced difference in RAGE expression was observed in EPCs. To further investigate the expression of RAGE protein in EPCs, cells were then incubated with 500 μg/L CML or CEL for 0, 24, 48, and 72 hours. CML and CEL did not increase RAGE protein expression in EPCs either (Figure 3).

### Effects of CML and CEL on MAPK activity

**Figure 4:**
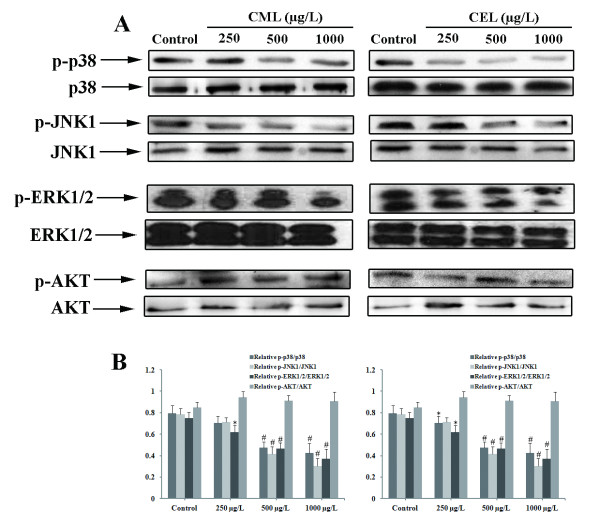
**Effects of CML and CEL on the MAPK and AKT signaling pathways in EPCs. **(**A**) After incubation of EPCs with different concentrations of CML or CEL for 24 hours, aliquots of cell lysate were subjected to western blot analysis. (**B**) The quantities of phosphorylated and total kinases were estimated using Quantity One software. CML and CEL inhibited phosphorylation of MAPKs, including ERK1/2, JNK1, and p38 MAPK, in a dose-dependent manner, but did not inhibit AKT. Similar results were obtained in three separate experiments. Data were expressed as mean ± SD. *p<0.05 vs. control, ^#^p<0.01 vs. control

The mitogen-activated protein kinases (MAPKs) and AKT signaling pathways have been shown to be involved in cellular proliferation. For these reasons, we investigated the activities of MAPK and AKT in EPCs treated with CML or CEL. Incubation of EPCs with CML or CEL for 24 hours down-regulated MAPK activity in a dose-dependent manner. The activity of ERK1/2, JNK1, and p38 MAPK were similarly down regulated in a dose-dependent manner. Treatment did not inhibit AKT activity (Figure 4).

## Discussion

In the past ten years, the understanding of endothelial dysfunction in CKD has greatly expanded. Important findings include the discovery of circulating EPC, reflecting endothelial repair capacity, and the demonstration of the specific toxicity of uremic compounds [13]. The relationship between circulating EPC levels and uremic toxins in CKD patients is not well understood [15].

AGEs have been identified in the blood and tissues of patients with ESRD [33]. Oxidative and carbonyl stress have been identified as factors in uremia [34]. Endogenous AGEs undergo cellular proteolysis and are released into plasma as AGE free adducts for urinary excretion [35]. These glycation free adducts include free fructoselysine (FL), free hydroimidazolones, CML, CEL, pentosidine, and other AGEs [35]. The normal high renal clearance of AGE free adducts is impaired markedly in CKD and thus considered as potential uremic toxins [17].

AGEs have been demonstrated to impair endothelial functions in several ways. They up-regulate adhesion molecule expression [36] and increase endothelial layer permeability [37]. *In vitro*, AGEs prompt intracellular generation of hydrogen peroxide and activation of nicotinamide adenine dinucleotide phosphate (NADPH) oxidase [38]. AGEs enhance apoptosis, depress EPC migration and tube formation in a concentration-dependent manner, and increase RAGE expression in these cells [18-20]. The negative impact of AGEs on bone marrow mesenchymal stem cells (MSCs) has been reported by us previously [39].

Mixtures of AGEs have been prepared for study after a long, complex, *in vitro* preparation process, resulting in “AGE-modified” proteins of unknown composition [40]. Few studies have used the complete range of genuine uremic AGE compounds. [Glorieux et al. 29] reported that genuine AGE compounds, including CML and CEL, could activate leukocyte response and hence play a role in uremia related atherogenesis. [Wang et al. 27] found that CML could accelerate progression of atherosclerotic calcification in diabetes. In the present study, the biologic effects of CML and CEL were studied, with specific emphasis on EPC function.

We believe the concentrations of CML and CEL used in this study requires special comment. EUTox reported the maximum concentration of CML is 6.9 mg/L in CKD patients in 2003 [19]. In their follow-up publication in 2007 [41], EUTox reported the maximum concentration (Cmax) of CML to be 111–221 nM (≈ 40 μg/L) and CEL to be 336–817 nM (≈ 200 μg/L) in CKD patients [23]. Recently, EUTox reported the Cmax of CML as 18.5 mg/L in CKD patients [24]. These different concentrations of CML and CEL might be explained by their use of different analytical methods, ELISA [17,24] and liquid chromatography-mass spectrometry (LC-MS) [41]. The quantification of AGEs using ELISA is problematic. Because of uncertain epitope specificity of antibodies employed, and the use of highly modified standard antigens dissimilar to the minimally modified antigens in physiological samples, AGE detection using ELISA does not usually provide AGE levels in absolute concentration, but rather in arbitrary units with or without normalisation to a reference AGE protein standard [42]. LC-MS analysis is now becoming the standard method for quantitation of glycation adducts [35]. We believe the findings published in 2007 were correct, according to our previous analysis (mean serum concentration of CML in ESRD patients without dialysis therapy ≈ 200 μg/L using LC-MS analysis, n = 5, data not published). For this reason we used CML and CEL in the 250–1000 μg/L range in this study. We believe previously published concentrations (2003, 2012) were higher than our study because of their use of ELISA to detect AGE.

MAPKs are a family of serine/threonine kinases comprised of extracellular signal-regulated kinase (ERK), c-Jun N-terminal kinase (JNK), and p38 MAPK (p38) [43]. Growth factor-induced proliferation of EPC has been demonstrated to be regulated by activation of the MAPK signaling pathway [44,45]. The MAPK signaling pathway activation has been demonstrated to be biphasic [46-50], with an early rapid increase in MAPK activation from 5 to 60 minutes, followed by a late second wave of MAPK activation of lower amplitude beginning at 7–10 hours. This second wave of MAPK activity was sustained for hours after growth factor stimulation and was critical for cellular progression from G1 into S phase [46,48,49,51]. In this study, the activity of MAPKs in EPCs treated with CML or CEL for 24 hours was tested. The reduced growth response to CML or CEL in EPCs was accompanied by significantly less phosphorylation of MAPKs, suggesting that CML and CEL may reduce EPCs proliferation via inhibition of the MAPK signaling pathway.

Our study found that CML and CEL had no effects on AGE receptor (RAGE) expressions in EPCs. This is consistent with previous studies, which found that CML-modified proteins were unable to bind to RAGE [52]. This may be because proteins modified by AGEs to a high extent (30–40 AGE residues per molecule) are competent AGE receptor ligands, whereas proteins modified by AGEs to a minimal extent (1–2 AGE residues per molecule) are not [53,54].

## Limitations

Though our results indicate that CML and CEL in uremic concentrations were less toxic on EPC functions, we did not study them together. The mechanism of toxicity of both CML and CEL will be the source of further study. Their *in vivo* effect on EPC function, like postnatal neovascularization, should also be studied.

We found that the anti-proliferative effects of CML and CEL on EPCs were associated with down-regulation of MAPK phosphorylation. Whether this could mediate inhibition of EPCs is still unknown. Signaling pathway inhibition experiments could be performed to confirm this hypothesis.

## Conclusions

It has not yet been determined which of the individual AGE uremic compounds exert toxic biologic effects [55]. In the present study, the effect of chemically well-defined AGE compounds, CML and CEL, on EPC functions was evaluated. Our study revealed that CML and CEL have low biological toxicity when separately tested. The biologic effects of AGE free adducts on the cardiovascular system merits further study.

## Abbreviations

AGEs: Advanced glycation end-products; BrdU: Bromodeoxyuridine; CEL: Nepsilon-(carboxyethyl)lysine; CKD: Chronic kidney disease; CML: Nepsilon-(carboxymethyl)lysine; CVD: Cardiovascular disease; ELISA: Enzyme linked immunosorbent assay; EPCs: Endothelial progenitor cells; ERK: Extracellular signal-regulated kinase; ESRD: End stage renal disease; EUTox Work Group: European Uremic Toxin (EUTox) Work Group; FACS: Fluorescence-activated cell sorting; FBS: Fetal bovine serum; HBSS: Hank’s balanced salt solution; HD: Hemodialysis; JNK: c-Jun N-terminal kinase; LC-MS: Liquid chromatography-mass spectrometry; MAPKs: Mitogen-activated protein kinases; MNCs: Mononuclear cells; MSCs: Mesenchymal stem cells; NADPH oxidase: Nicotinamide adenine dinucleotide phosphate oxidase; PBS: Phosphate buffered saline; PD: Peritoneal dialysis; RAGE: Receptor for advanced glycation end products; VEGF: Vascular endothelial growth factor.

## Competing interests

The authors declare that they have no competing interests.

## Authors’ contributions

JZ and KY carried out the cell culture experiments and drafted the manuscript. The molecular studies were performed by YJ. ZZ and RD were involved in the interpretation of the results. RZ, the correspondence author, has contributed enormously to devising the entire study plan and supervising the entire process. Prof. LL helped greatly with editing the presented manuscript. All authors have read and approved the final manuscript.
